# Effectiveness of Visual Methods in Information Procedures for Stem Cell Recipients and Donors

**DOI:** 10.4274/tjh.2016.0118

**Published:** 2017-12-01

**Authors:** Çağla Sarıtürk, Çiğdem Gereklioğlu, Aslı Korur, Süheyl Asma, Mahmut Yeral, Soner Solmaz, Nurhilal Büyükkurt, Songül Tepebaşı, İlknur Kozanoğlu, Can Boğa, Hakan Özdoğu

**Affiliations:** 1 Başkent University Adana Application and Research Center, Department of Biostatistics, Adana, Turkey; 2 Başkent University Faculty of Medicine, Department of Family Medicine, Adana, Turkey; 3 Başkent University Faculty of Medicine, Adana Adult Bone Marrow Transplantation Center, Adana, Turkey

**Keywords:** Hematopoietic stem cell, Donor, Informed consent, Audiovisual method, Bone marrow transplantation

## Abstract

**Objective:**

Obtaining informed consent from hematopoietic stem cell recipients and donors is a critical step in the transplantation process. Anxiety may affect their understanding of the provided information. However, use of audiovisual methods may facilitate understanding. In this prospective randomized study, we investigated the effectiveness of using an audiovisual method of providing information to patients and donors in combination with the standard model.

**Materials and Methods:**

A 10-min informational animation was prepared for this purpose. In total, 82 participants were randomly assigned to two groups: group 1 received the additional audiovisual information and group 2 received standard information. A 20-item questionnaire was administered to participants at the end of the informational session.

**Results:**

A reliability test and factor analysis showed that the questionnaire was reliable and valid. For all participants, the mean overall satisfaction score was 184.8±19.8 (maximum possible score of 200). However, for satisfaction with information about written informed consent, group 1 scored significantly higher than group 2 (p=0.039). Satisfaction level was not affected by age, education level, or differences between the physicians conducting the informative session.

**Conclusion:**

This study shows that using audiovisual tools may contribute to a better understanding of the informed consent procedure and potential risks of stem cell transplantation.

## Abstract (tr)

**Amaç:**

Hematopoietik kök hücre alıcıları ve donörlerden bilgilendirilmiş onam alınması, nakil sürecinin en önemli basamağıdır. Görsel yöntemlerden yararlanılması anlamayı kolaylaştırabilir. Bu prospektif randomize çalışmada sözel ve yazılı bilgilendirmeye ilave olarak audiovizüel yöntem kullanılmasının standart yönteme göre etkinliğinin araştırılması amaçlanmıştır.

**Gereç ve Yöntemler:**

On dakikalık kısa bir bilgilendirme animasyonu hazırlatıldı. Toplam 82 katılımcı rastgele 2 gruba ayrıldı: Grup 1 (görsel bilgilendirme yönteminin ilave edildiği grup) ve grup 2 (standart yöntem uygulanan grup). Bilgilendirme işlemi sonunda katılımcılara 20 soruluk bir anket uygulandı. Ayrıca, yeni yöntemin sonuçlarının kişiler arası farklılıktan etkilenip etkilenmediği test edildi.

**Bulgular:**

Güvenirlik testi ve faktör analizi anketin güvenilir ve geçerli olduğunu gösterdi. Tüm olgular için genel memnuniyet ortalama değeri 200 üzerinden 184,8±19,8 olarak bulundu. Yazılı bilgilendirilmiş onam form memnuniyeti ortalama puanları yönünden grup 1’deki olguların memnuniyetleri grup 2’ye oranla anlamlı olarak daha yüksek olduğu saptandı (p=0,039). Sözel olarak bilgi veren doktor ve bilgilendirme animasyonu memnuniyeti bakımından gruplar arasında fark saptanmadı. Olguların memnuniyet seviyesi yaş, eğitim durumu ve bilgilendiren kişiler arası farklılıktan etkilenmedi.

**Sonuç:**

Bu çalışma, görsel yöntemlerin kullanılmasının işlem ve muhtemel risklerinin daha iyi anlaşılmasına katkı sağlayabileceğini göstermektedir.

## INTRODUCTION

Stem cell transplantation (SCT) is a procedure with severe morbidity and mortality, but it also has the potential for long-term survival and recovery [1]. An informed discussion with the patient and his or her relatives and a comprehensive examination of the patient and the donor that includes psychosocial aspects are central to pre-transplant preparation [2]. SCT cannot be performed without collaboration with the patients and their relatives, as treatment may result in short- and long-term changes that affect the patient’s life. Therefore, patient contribution is essential for a detailed educational discussion and provision of informed consent [3,4,5,6,7].

The rationale, procedure, and potential outcomes for SCT can be difficult to understand [1,3]. As the patient may have severe anxiety due to an often difficult diagnosis and potentially fatal outcome, it is unrealistic to expect the patient to easily understand this information. Therefore, to overcome this difficulty, transplant doctors have developed their own communication methods based on personal experiences.

The reason for SCT is often not clear to donors and recipients, and the benefits and drawbacks of transplantation may need to be discussed in detail. The correct timing for transplantation is another issue. In many situations, transplantation may be postponed until other therapeutic methods are attempted [3]. Once transplantation becomes feasible, both short- and long-term adverse events are discussed. Patients are informed clearly and objectively about potential side effects. The possibility of procedure-related death and other severe conditions (e.g., admission to the intensive care unit or life support) are also discussed [7]. Requirements for interventional procedures to evaluate potential side effects are also covered. Non-fatal side effects (e.g., chronic graft-versus-host disease) are mentioned as possible long-term effects. Informed consent is only obtained after this information is clearly communicated in accordance with laws, regulations, and standards [7].

The goal of patient and donor education is to help them understand and accurately evaluate the information and risks. Therefore, there needs to be verification of patient and donor understanding throughout the educational procedure for SCT [7]. However, there are a limited number of reports describing the effectiveness of visual methods in patient/donor education about SCT.

On this study, we investigated the effectiveness of an informational animation for transplant patients and donors in pre-transplant education.

## MATERIALS AND METHODS

### Study Design

This study was conducted between June 2013 and July 2014 using a prospective, randomized, cross-sectional single-center design. The sample comprised adult patients who were scheduled to undergo autologous or allogeneic peripheral SCT at the Adana Bone Marrow Transplantation Unit of Başkent University Faculty of Medicine and donors from whom peripheral stem cell collection for allogeneic transplantation was planned. The standard operating procedure (SOP: KIT-KU 005) was applied to patients and donors after the council decision had been obtained for transplantation and cell collection, in accordance with JACIE standards. The transplant coordinator invited donors and patients to participate in the study. The clinical medical director, transplant doctor, and transplant coordinator also participated in the informational meeting, and a transplant nurse participated when necessary. Donors were asked to attend the session alone in accordance with the donor privacy principle. However, patients could request that first-degree relatives attend the session with them.

Participants were randomly selected and divided into two groups. Group 1 was the study group, exposed to audiovisual information in addition to standard verbal and written information. Group 2 was the control group and received only standard verbal and written information. The transplant coordinator obtained feedback from participants and completed a questionnaire that measured the quality of the information session. To eliminate ethical problems, the audiovisual information was provided for group 2 (control group) after their initial feedback on the information session. The verbal information in the sessions was delivered by two separate transplant doctors to test whether the audiovisual method was affected by interpersonal differences.

The results were evaluated in accordance with the rules stated in the Clinical Trials section of the JACIE standards by the Study Board of the Başkent University Adana Bone Marrow Transplantation Unit. Approval was obtained from the Başkent University Scientific Research Board.

### Verbal and Written Information

Both groups received standard verbal and written information. This covered disease status, purpose of the treatment, treatment principles, stem cell collection procedure, pretreatment assessment, the drugs used and their side effects, infusion of stem cells, benefits expected from the treatment, treatment risks and side effects, other treatment options, and disposal of the cellular product. These topics were prepared locally in accordance with international standards (FACT-JACIE standards) to meet donor and recipient information requirements. Patients and donors were able to ask questions after the information session had been completed [4,5,6,7]. The information session lasted up to 30 min.

### Information Animation

The information animation was based on the flow of the topics discussed in the verbal and written information session. Some topics were covered in movie format and others were shown as graphics and images. A Three D Studio Max program used to prepare the program and the technical support was provided by a technical company (Teknik Medya, Adana, Turkey). The visual animation lasted 10 min, and there were Turkish, Arabic, and English language and caption options.

### Obtaining Patient and Donor Feedback

A 20-item questionnaire was used to collect feedback including demographic data (age, sex, education status, job, and the institute where the patient was first diagnosed). In addition, a 20-item scale was prepared to measure participants’ satisfaction. Seven items assessed satisfaction with the written informed consent form, seven items were about the information provided by the doctor, and six items focused on the audiovisual information. After an interactive interview, participants scored each question from 1 to 10 based on their satisfaction level. The questions were pre-tested with 10 randomly selected healthy subjects before the study to confirm intelligibility. The scores of the 20 questions were summed to give the overall satisfaction level. The seven questions concerning the written informed consent tested satisfaction with the information on the informed consent form regarding issues such as side effects, stages of treatment, and treatment method. Questions about the doctor who provided the information evaluated the same issues. The questions measuring satisfaction with the information animation also evaluated how the patient understood the stages of the disease and the treatment process.

### Statistical Analysis

Statistical analysis was performed with SPSS 17.0. Categorical measurements were summarized as number and percentage and continuous measurements as mean and standard deviation (median and minimum - maximum where needed). Chi-square or Fisher’s exact tests were used for comparison of categorical variables. The inter-rater agreement was analyzed with kappa statistics. The consistency between questions was evaluated using Cronbach’s alpha coefficient. The value of Cronbach’s alpha coefficient reflects the reliability and internal consistency of the scale (<0.40 indicates that a scale is not reliable, 0.40 to <0.50 indicates very low reliability, 0.50-0.60 low reliability, 0.60-0.70 sufficient reliability, 0.70-0.90 high reliability, and ≥0.90 very high reliability). The reliability of the scale was tested with factor analysis. The appropriateness of the data structure for factor analysis was evaluated with the Kaiser-Meyer-Olkin (KMO) test, where <0.50 indicates that factor analysis could not be continued, 0.50 and 0.60 are interpreted as poor, 0.60-0.70 as weak, 0.70-0.80 as moderate, 0.80-0.90 as good, and above 0.90 as excellent. Bartlett’s test of sphericity was used to test the association between statements.

Distributions were controlled for inter-group comparisons. Student’s t-test was used for variables showing parametric distribution, and the Mann-Whitney U test was used for variables not showing parametric distribution. A p-value of less than 0.05 was considered statistically significant

## RESULTS

In total, 92 subjects who were scheduled to undergo autologous or allogeneic hematopoietic peripheral SCT and peripheral stem cell donors for allogeneic transplantation were invited to participate in the study. Of these, 82 (89%) agreed to participate, and 41 participants were assigned to each group. The ten individuals who did not agree to participate were allogeneic SCT recipients. The mean age of participants was 47±14 years (range: 15-67). Mean age of both group 1 and group 2 was 47±14 years (p=0.886). Nine participants (11%) were sibling donors, all in group 1.

Participants’ demographic characteristics are shown in Table 1. No statistically significant differences were found between groups in terms of sex, marital status, educational status, center where their diagnosis was made, and patient diagnoses (p>0.05, for all).

Before starting the reliability and validity analysis, the present researchers reviewed the questionnaires to determine the participants who had repeated the same answer. This was not found to be a significant problem, and analysis continued with 82 participants. The questionnaire used to determine patient satisfaction had a Cronbach’s alpha coefficient of 0.94 (95% confidence interval [CI] 0.92-0.96), indicating that it was highly reliable.

We used factor analysis to measure the validity of the scale. The 20-item satisfaction scale comprised three parts. Consistency of data in factor analysis was measured with KMO sample sufficiency and Bartlett’s test of sphericity. The KMO value was 0.769 and the Bartlett’s test results were statistically significant (χ^2^=2216.4, p=0.0001). The results of both tests showed that factor analysis of satisfaction scale data was appropriate.

In factor analysis of the 20-item satisfaction scale, questions with sample adequacy below 0.50 were investigated. No questions were eliminated because no statement showed a factor load below 0.50. Factor analysis detected three factors, all of which had eigenvalues of ≥1, and there were no overlapping expressions. Factor 1 comprised seven items and explained 25.6% of the total variance, factor 2 explained 15.4% of the variance, and factor 3 explained 25.7%. The total variance explained by three factors was 66.7% (Table 2).

The Cronbach’s alphas for the satisfaction scale were 0.95 for written consent, 0.91 for the informing doctor, and 0.90 for the informational animation. This indicates that all sections were highly reliable.

The first physician conducted information sessions for 41 participants (21 in group 1, 20 in group 2). The second physician conducted the information sessions for the remaining 41 participants (19 in group 1, 22 in group 2). Both physicians had 10 years of experience as transplant physicians.

**Figure 1:**
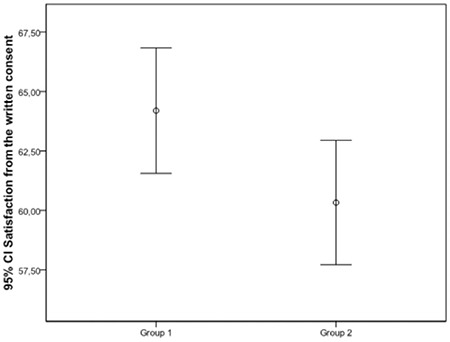
Figure 1. Patient satisfaction with the written consent. CI: Confidence interval.

The Cronbach’s alpha for participants’ satisfaction with the patient/donor information session was 0.94 (95% CI 0.92-0.96). Table 3 shows participants’ satisfaction with the information session. Satisfaction with the written informed consent, the informing doctor, and the informational animation was measured with an overall satisfaction level that was high (184.8±19.8) compared with the maximum value of 200. There was no significant difference between groups with regard to overall satisfaction. However, a statistically significant difference was found between groups for satisfaction with the written informed consent form. Patient satisfaction was greater in group 1 (p=0.039) (Table 3; Figure 1). There were no significant differences between groups for satisfaction with the doctor who provided the information and the informational animation. The level of satisfaction was not affected by sex or educational status in either group (p>0.05 for all).

## DISCUSSION

This study investigated the effectiveness of audiovisual information in providing essential information during the informed consent process for patients and donors. There were differences in patient/donor satisfaction between the session that included audiovisual information and that comprising standard verbal/written explanations. To our knowledge, this is the first study to investigate the effects of audiovisual materials in providing information to hematopoietic stem cell recipients and donors.

In general, the use of audiovisual materials facilitates learning and reduces learning time [8,9]. This observation has been supported physiologically [10,11] and is reminiscent of the Chinese saying “I forget if I hear, I remember if I see, I learn if I do”. In this context, our study aimed to develop an information technique using audiovisual methods and demonstrate its efficacy with a verification study in the context of an important issue such as bone marrow transplantation and stem cell donation.

Hematopoietic SCT is an effective treatment that is performed for many life-threatening diseases; however, it may result in significant morbidity and mortality [3]. International standards and national laws and regulations require that informed consent be obtained from bone marrow recipients and bone marrow and stem cell donors [7]. These individuals are informed about the rationale of the procedure, expectations, application technique, potential difficulties, and other options if the procedure is not approved. Verbal and written information is provided. The main goal of this information is to enable the subject to make accurate risk assessments and provide informed consent. This is related to correct understanding of the information [12].

A standard operating procedure was produced for the information methods used in this study. In this procedure, we determined the information field, the individuals responsible for patient/donor information, the individuals who would attend the formal meeting, national laws and regulations, and the information required for FACT-JACIE standards [7]. The duration of the informational session and the materials used in the session and consent process were standardized. Approval was obtained from parents or custodians for subjects aged <18 years and those who were not able to give consent (e.g., disabled subjects). A special arrangement was made for pediatric patients or donors (i.e. a psychiatrist joining the interview).

We found no statistically significant difference between groups for overall satisfaction. However, satisfaction with the written informed consent form was greater in the study group compared with the control group. Participants in the study group answered questions about treatment stages after sequentially being given written, verbal, and video explanations, and they were satisfied with the information given about their disease. However, the control group provided initial feedback after the written and verbal information session and additional feedback after they viewed the animation. Our results indicated that the video helped patients obtain a more accurate understanding of their disease.

Current FACT-JACIE standards (version 6.0) do not require visual materials in addition to verbal and written information for hematopoietic stem cell recipients and donors [7]; verbal and written information is considered sufficient. However, the results of our study support the theory that there are benefits to providing information using audiovisual materials, such as a decrease in perception difficulty arising from language and cultural differences, intellectual differences, and aging.

In the present study, participants in both groups evaluated their experiences of the physician who conveyed information as similar, which may be regarded as a limitation of the study. However, as bone marrow transplantation is a critical issue, it is likely that doctors use similar statements to convey essential information. 

## CONCLUSION

In conclusion, using audiovisual materials and standard methods for providing information to bone marrow recipients and donors may positively affect patient/donor perception and overcome unnecessary anxiety. Although we obtained data about patient/donor satisfaction at the time of providing informed consent, further studies may reveal whether better understanding of the transplant/donation procedure would result in better transplant/donation experience and outcomes.

## Figures and Tables

**Table 1:**
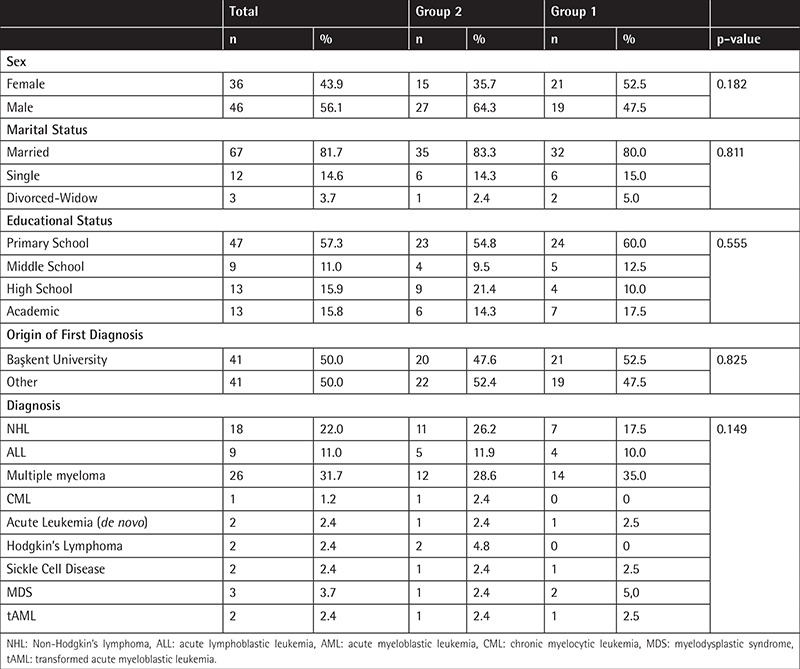
Characteristics of responder patients at the time of the information.

**Table 2:**
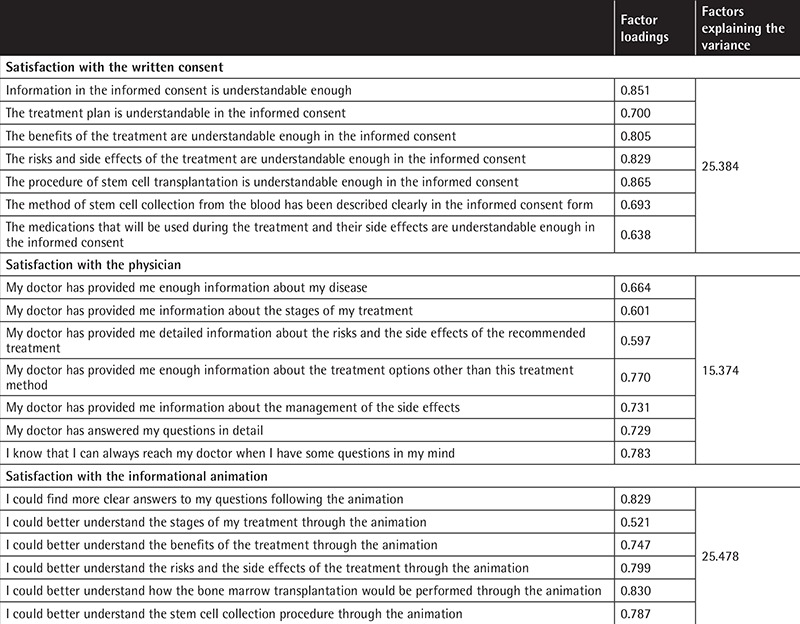
Factor analysis.

**Table 3:**
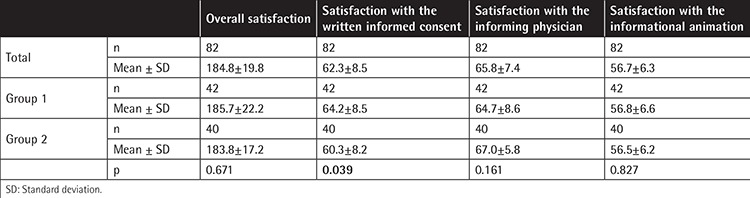
Participant satisfaction.
